# The difference in the elderly’s visual impact assessment of pocket park landscape

**DOI:** 10.1038/s41598-023-43522-y

**Published:** 2023-10-06

**Authors:** Xinyu Wang, Guanjun Li, Jingyin Pan, Jiamin Shen, Chenping Han

**Affiliations:** China University of Mining and Technology, No. 1, Daxue Road, Xuzhou City, Jiangsu Province China

**Keywords:** Forestry, Urban ecology, Geriatrics, Quality of life, Civil engineering

## Abstract

As an important part of urban public open space, pocket parks have become an important activity place for the elderly in the context of the aging society in China. With the pocket parks in Nanjing, Jiangsu province, China as research object, this paper set six landscape features to be studied, namely, Height of trees, Green color richness, Stratification of green landscapes, Green space ratio, Leisure facilities, and Water landscape. The elderly respondents with different demographic characteristics, such as age, gender, education level and residential type, were subjected to the picture stimulation experiment whose results were then statistically analyzed. The results indicate that gender and residential type exert certain influence on the elderly’s visual impact assessment of pocket park landscape. To be specific, the male elderly prefer the pocket park landscape with 3-6 m high trees, medium green space ratio, and more leisure facilities; the female elderly are in greater favor of pocket park landscapes with 0-3 m high trees, five or more colors, three or more layers; the elderly who live with their families prefer pocket park landscapes with medium green space ratio and more leisure facilities; to the elderly who live alone, pocket park landscapes with trees which are 0–3 m high, five or more colors, and medium leisure facilities are more attractive. This study can provide valuable reference for pocket park design in China.

## Introduction

### Pocket park landscape

Pocket parks are small-scale urban open spaces dotted throughout high-density urban areas. Generally, the area of a pocket park is no larger than 3000 m^2^^1^. Pocket parks are usually adapted from leftover space in cities. For example, Paley Park was constructed by Robert ZernMarshall^2^ from the space between high-rise buildings^2^. Compared with large-scale parks or greenbelts, pocket parks are small in scale and widely distributed, which meets the demand of urban residents for daily contact with nature^3^. In this sense, pocket parks are endowed with ecological, social, and landscape functions. Pocket park landscapes are usually composed of paved sites, seats, arbours, fitting facilities, plant landscapes, and waterscapes^4^.

China has entered an ageing society, and elderly people have become an important group in society. They cannot go to large parks that are far away due to physiological reasons, so pocket parks have become important places for leisure activities for elderly people^5^. A good pocket park landscape is of great help for elderly people to rest, enjoy beautiful scenery, and promote their physical and mental recovery. Pocket parks can sustain the capability of elderly people to live independently and participate in social interactions.

Many scholars have conducted research on pocket parks and pocket park landscapes. Roy, et al.^6^ observed that elderly people aged 66 to 75 were more frequent visitors to pocket parks than other age groups. Kerishnan, et al.^3^ found that people with partners were more inclined to visit pocket parks. Nordh, et al.^1^ conducted a related study and showed that pocket park landscapes could improve mental recovery. Amp and Tynonsupa/Sup^7^ concluded that pocket park landscapes with better afforestation encouraged people to use outdoor space and thus promoted social interaction. However, studies on pocket park landscapes, especially greening, are still rare.

### Visual impact assessment

Visual impact assessment is a widely used method of assessing the impact of various landscape elements on humans’ visual perception. Lin, et al.^8^ discovered that the building height and vegetation types around lakes influence people’s visual assessment of them. Tveit^9^ maintained that the assessment of landscape characteristics based on respondents’ mentality is an indispensable part of landscape visual impact assessment. In an investigation of the visual impact assessment of landscapes, Shuttleworth^10^ claimed that landscapes possess internal or external beauty, and Kaplan and Kaplan^11^ noted that this beauty could be quantified by certain dimensions. Visual impact assessment plays an important role in people’s lives and is a reliable way to improve the quality of landscapes via design and management^12^.

In terms of studies on urban green space landscapes, some researchers have used pictures to replace actual landscapes in studies of visual impact assessment^13–15^. In these studies, respondents assess pictures rather than actual landscapes. Wang and Zhao^16^ employed the method of visual impact assessment to study residents’ visual preference for urban greenbelt vegetation landscapes in Xuzhou, Jiangsu Province, China. Their study revealed that naturalness, the growth status of plants, and elements other than plants exerted an impact on male residents’ visual preference for landscapes, while the degree of plant maturation and number of colours were highly influential for female residents’ visual preference. Ng, et al.^14^ used pictures to explore the public’s cognition of urban plants and preference for street elements. Although studies on visual impact assessment are not rare, visual impact assessment of elderly people has not been emphasized in academia.

### Demographic differences in elderly people

Daniel^17^ and Sevenant and Antrop^18^ maintained that due to the influence of cognitive motivation, people with different social backgrounds have variations in terms of landscape preference. According to a study conducted by Gonzalo and Mühlhauser^19^, these variations exist but are not significant. Tveit^9^ claimed that demographic characteristics influence people’s visual impact assessment.

According to the “Law of the People's Republic of China on the Protection of the Rights and Interests of the Elderly”, the minimum age threshold for elderly people is 60 years old. Therefore, the research object in this study was defined as elderly people over 60 years old who had the ability to take care of themselves. In previous studies, the demographic characteristics of elderly people mainly included education level^20^, age^21^, gender^22^, and type of residence^23^. Accordingly, this study used these four demographic characteristics of elderly people to explore the visual impact assessment of elderly people on the features of pocket park landscapes.

### The features of pocket park landscapes

Many factors may influence elderly people’s visual impact assessment of pocket park landscapes. Among these, the demographic characteristics of elderly people and the landscape features of pocket parks cannot be neglected^16^. Pocket park landscapes are composed of various elements, such as afforestation^24^, leisure facilities^25^, and water landscapes^26^. These elements may directly influence the visual impact assessment of elderly individuals.

The afforestation of pocket parks influences people’s visual impact assessment. A study conducted by Sarah, et al.^27^ indicated that tall trees that could offer shade in urban green space landscapes would positively attract elderly people to perform physical exercise in the park, which in turn would render a higher visual impact assessment for elderly individuals. The number of colours and diversity of pocket park landscapes can also enhance the beauty and improve the quality of the whole landscape, which generates a desirable visual impact assessment among respondents^28^. Ying, et al.^29^ observed that in general, a highly layered plant community wins the approval of most respondents. In addition, hard landscapes and park green spaces have a large effect on people’s mental recovery^30^. Elderly people are likely to walk on hard paved squares where the green space ratio is relatively low. However, due to the decline in their physiological function, some elderly people prefer soft ground, such as lawns, where the green space ratio is high.

Leisure facilities also influence people’s visual impact assessment. Kaczynski, et al.^31^ claimed that seats should be provided in parks to satisfy people’s demand for social gathering and interaction. According to an Australian study, over 70% of elderly people prefer parks with seats^32^. Cohen, et al.^33^ proposed that the installation of fitting facilities could also increase the attraction of parks. Abdelhamid and Elfakharany^25^ maintained that shading devices could reduce air temperature and improve the comfort level in summer, thus creating a comfortable park environment for elderly individuals. Similarly, shading devices may make elderly people more likely and willing to take walks. The installation of leisure facilities exerts a positive influence on the choices of elderly people^34^, in line with an investigation conducted by Abdelhamid and Elfakharany^25^.

Water landscapes are also influential to people’s visual impact assessment. As an element with high ecological value, water can activate a space and make landscapes in the park more amiable. William H. Whyte, an American sociologist, maintained that water was the key element for any popular urban space. Masoudi^35^ claimed that water could mitigate the urban heat island effect. As demonstrated by many studies, water landscapes are the most psychologically restorative landscapes^36^. Water is also considered a key landscape attribute that can stimulate the recovery of elderly people^26^. Most elderly people feel annoyed by traffic noise^37^, but the sound of water emitted by waterscape facilities can effectively reduce noise interference. Hamia, et al.^15^ found that water landscapes were the most preferred type of landscape, especially during the hot summer. Elderly people prefer to visit pocket parks with water landscape facilities and linger around them^38^.

Based on the classification of elderly people’s demographic characteristics, six landscape features are summarized in consideration of their influence on elderly individuals’ visual impact assessment of pocket park landscapes. The six features are the height of trees, green colour richness, the stratification of green landscapes, green space ratio, leisure facilities, and water landscapes. Specific values are assigned to these features (as shown in Table 1), which can provide a reference for the design of pocket park landscapes. The six landscape features summarized and adopted in this study were obtained by analysing the landscape features recognized by other scholars and the characteristics of the studied landscapes^39–43^. The method of summarizing the characteristics of research objects through literature research is widely used in similar papers^5,44^.

**Table 1:** Measurement scale of pocket park landscape features.

Landscape characteristic	Scoring		
Height of trees	0–3 m = 1	3–6 m = 2	6–12 m = 3
Green colour richness	One or two = 1	Three or four = 2	Five or more = 3
Stratification of green landscapes	One layer = 1	Two layers = 2	Three layers and above = 3
Green space ratio	Low = 1	Medium = 2	High = 3
Leisure facilities	None = 1	A few = 2	More = 3
Water landscape	None = 1	A few = 2	More = 3

### Research questions

In previous studies on people’s views of or satisfaction with parks, external factors such as convenience, accessible transportation, social connection, and mobility were included in the statistical model as the main influencing factors; however, internal factors such as landscape features have often been neglected^45^. Little research has investigated elderly people’s visual impact assessment of pocket park landscapes. Therefore, pictures were used to replace actual pocket parks in this study so that the respondents’ focus would be on the internal factors of pocket parks. This study attempts to fill the research gap regarding the landscape features of pocket parks.

A questionnaire survey was conducted to collect the demographic characteristics of elderly respondents and their visual impact assessment of specific pocket park landscapes. Through data statistics and analysis of the assessments and demographic characteristics, this study aimed to answer the following two questions:

How do landscape features influence elderly people’s visual impact assessment of pocket park landscapes?

For elderly people with different demographic characteristics, is there any difference in their visual impact assessment of pocket park landscapes? If yes, in what way?

## Research method

### Investigation site

**Figure 1:**
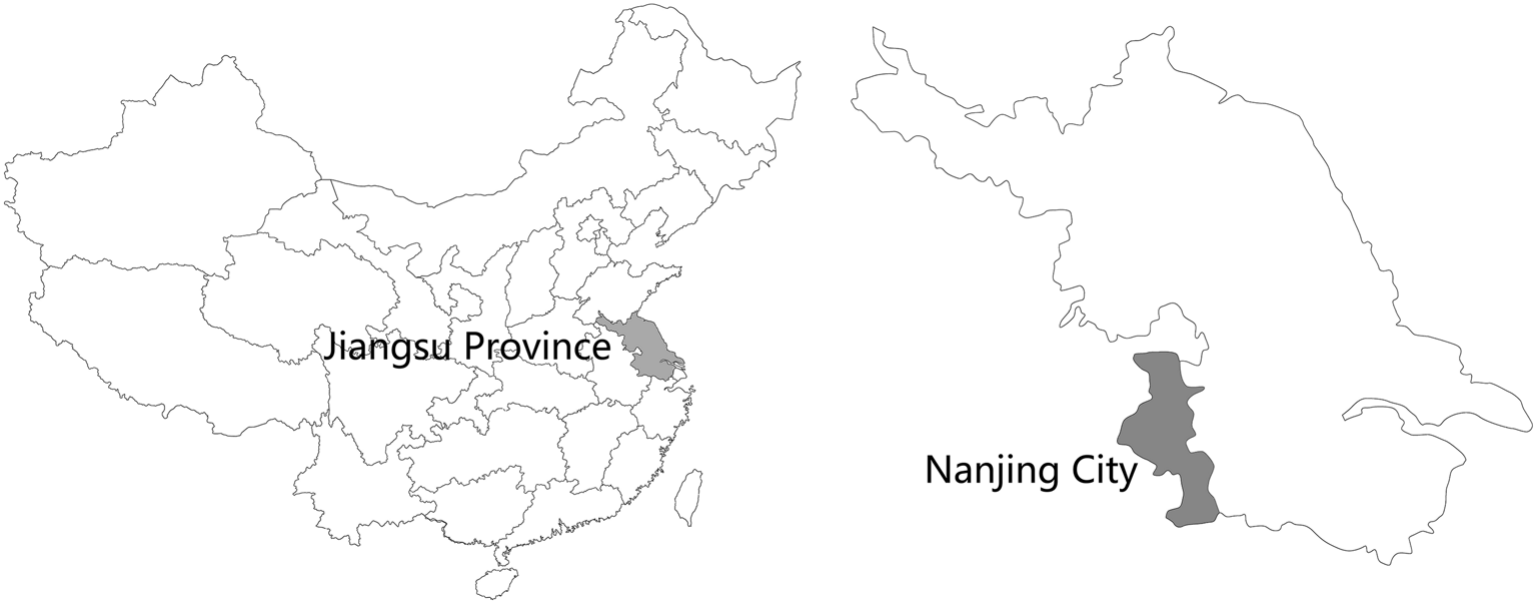
Map of Nanjing, Jiangsu Province, China. (Basemap image come from OpenStreetMap-2021–05-06, https://www.openstreetmap.org/. This figure is edited and generated by Adobe Photoshop 2021 software).

The investigation site of this experiment was Nanjing, Jiangsu Province, China (Fig. 1). Nanjing, located in the middle and lower reaches of the Yangtze River, has a humid climate in the northern subtropical region with four distinct seasons and abundant rainfall. The main vegetation type is broad-leaved evergreen deciduous forest^46^. Nanjing has a highly developed economy and a high level of urbanization. It is also a national ecological city. Nanjing has entered an “advanced ageing society”, with registered elderly people aged 60 or above accounting for 21.1% of the total population (Nanjing Statistics Bureau, 2019). Therefore, with Nanjing as its investigation site, this study is highly representative.

### Photo shooting

The experimental photos were taken by the author in Nanjing in March 2019. The locations selected were Gulou District, Qinhuai District, Xuanwu District, and Jianye District. The experimental scenes were set in popular pocket parks within each district. These four districts are all central urban areas of Nanjing, with a large number of elderly population.. The angle of view was the normal field of view of the photographer (about the vertical height 165cm, according to the report on Nutrition and chronic Diseases of Chinese residents released by the State Council in 2017, the average height of men and women in Jiangsu Province is 168 cm).The photos were taken from 10:00 to 4:00 pm in sunny or cloudy weather to control the lighting conditions. The equipment used for shooting was a Nikon D3100 digital SLR camera with a focal length of 18–55 mm. A total of 220 photos were taken and collected in the experiment, which were categorized into different groups based on the similarity of the stimulating factors. The team of landscape architects then selected the photos with fewer interfering factors from these groups to conduct the experiment^15^. As a result, only 24 photos were chosen in the questionnaire. The photos were used to evaluate visual preferences instead of real scenery, a method that has been widely used in previous studies^8,47^.

### The classification and judgement of pocket park landscape features

The value of variables denoting six different Landscape characteristics in Tables 1, selected according to above-mentioned literature, were determined by 10 professionals^13,15,48,49^. Using the Delphi method, we invited three architects, five landscape designers, and two forestry experts to evaluate and assess the value of each variable listed in Tables 1, respectively. The 24 photos plus one e-questionnaire were emailed to the ten professionals who were asked to complete the questionnaire independently.

Technical terms were used when the characteristics (as listed in Tables 1 ) were classified so that the professionals could evaluate the characteristics more carefully and accurately^16^.

### Investigation of the respondents’ visual impact assessment

In this study, photos were used in the questionnaire survey as a substitute for real scenery. This method has been widely applied in previous studies and has been proven to be effective^50^. Although photo display has certain limitations^17,51,52^, it is the most frequently used and most effective method for aesthetic assessment^17,53^.

The 24 selected photos were printed in full colour on paper in A4 format. To make it easier for people to rate the photos and to take into account the patience of the elderly interviewees, the 24 photos were printed on a total of four sheets of A4 paper and bound in a random sequence. The 24 photos were displayed in parks and squares (Including some of pocket parks where the photos were taken) in Nanjing, Jiangsu Province, where elderly people gather in large numbers, and randomly selected elderly respondents were invited to rate them. During the experiment, participants were free to flip through the printed paper and to review or change the scores of photos. In previous studies, pictures have been widely used to replace the actual landscape^54^ as the basis for the evaluation and judgement of visual preferences based on pictures.

The respondents were first asked to provide their demographic characteristics according to the questions on the questionnaire. This experiment included four demographic characteristics, namely, gender, age, education level, and residential type (Table 2). The respondents then rated the photos according to their own visual preference. Their ratings ranged from 1 to 5, with 1 denoting the lowest and 5 denoting the highest. The rating implications are shown in Table 3. The questionnaire survey was conducted from October to November 2019. In total, 358 respondents were surveyed and 297 valid questionnaires were collected, with an effective rate of 82.9%. The demographic characteristics of the elderly respondents are displayed in Table 4. As the statistics show, the demographic distribution of the elderly respondents was similar to that obtained by the Nanjing Bureau of Statistics (2018), supporting the representativeness of this study.

**Table 2:** The variables and set values of demographic characteristics.

Demographic characteristics	Variable	Set value
Gender	Female	1
	Male	2
Age	60–69 years old	1
	70–79 years old	2
	80 years old and above	3
Education level	Illiteracy	1
	Primary school	2
	Middle school	3
	College degree or above	4
Residential type	Live alone	1
	Live with family	2

**Table 3:** The implication of the ratings.

Rating	Implication
1	Dislike
2	Mildly dislike
3	Neutral
4	Mildly like
5	Like

**Table 4:** The demographic statistics of the elderly respondents.

Demographic characteristics	Variable	Number of respondents	Percentage	Percentage of Nanjing population (%)
Gender	Female	156	52.5	50.3
	Male	141	47.5	49.7
Age	60–69 years old	177	59.6	55.2
	70–79 years old	91	30.6	30.7
	80 years old and above	29	9.8	14.1
Education level	Illiteracy	39	13.1	14.8
	Primary school	70	23.6	23.6
	Middle school	164	55.2	54.2
	College degree or above	24	8.1	7.4
Residential type	Live alone	172	57.9	
	Live with family	125	42.1	

### Ethics statement

The topic was not ethically sensitive and was conducted in accordance with national and institutional legal and ethical requirements. Data were collected completely anonymously (i.e., no possibility of identifying the respondents). Therefore, this work falls outside the scope of GDPR 2016 and MDSM (Measures for Data Security Management) for China.

The project followed institutional guidelines and was discussed with the internal ethics reference person, who indicated that there is no need for ethical approval when surveys are not directly health related. In China, there is no legal requirement for ethical approval of such a survey when no sensitive issues are explored and no privacy is involved, and there are no IRB mechanisms in place for this type of work. Sensitive data or research involving human subjects undergoes ethical approval through ethical research committees based in hospitals that do not assess this type of project.

Additional ethical concerns were assessed internally. Participation was voluntary, and all participants were informed that the survey was anonymous and that all data would only be used for research and evaluated anonymously. To ensure privacy, all data were collected and analysed anonymously with no collection of identifiers/codes.

### Data analysis method

The collected data were analysed with SPSS 22.0. First, one-way ANOVA was conducted to examine the influence of demographic variables on the respondents’ visual preference for the pocket park landscape. Then, correlation analysis was performed to study the correlation among the demographic variables of the elderly respondents. On this basis, stepwise multiple linear regression analysis was conducted to explore the quantitative relationship between demographic characteristics and visual impact assessment and between the landscape features of pocket parks and the ratings given by different elderly groups. These analysis methods are commonly used in similar studies^16,55^.

## Results

### The overall assessment of the photos

**Figure 2:**
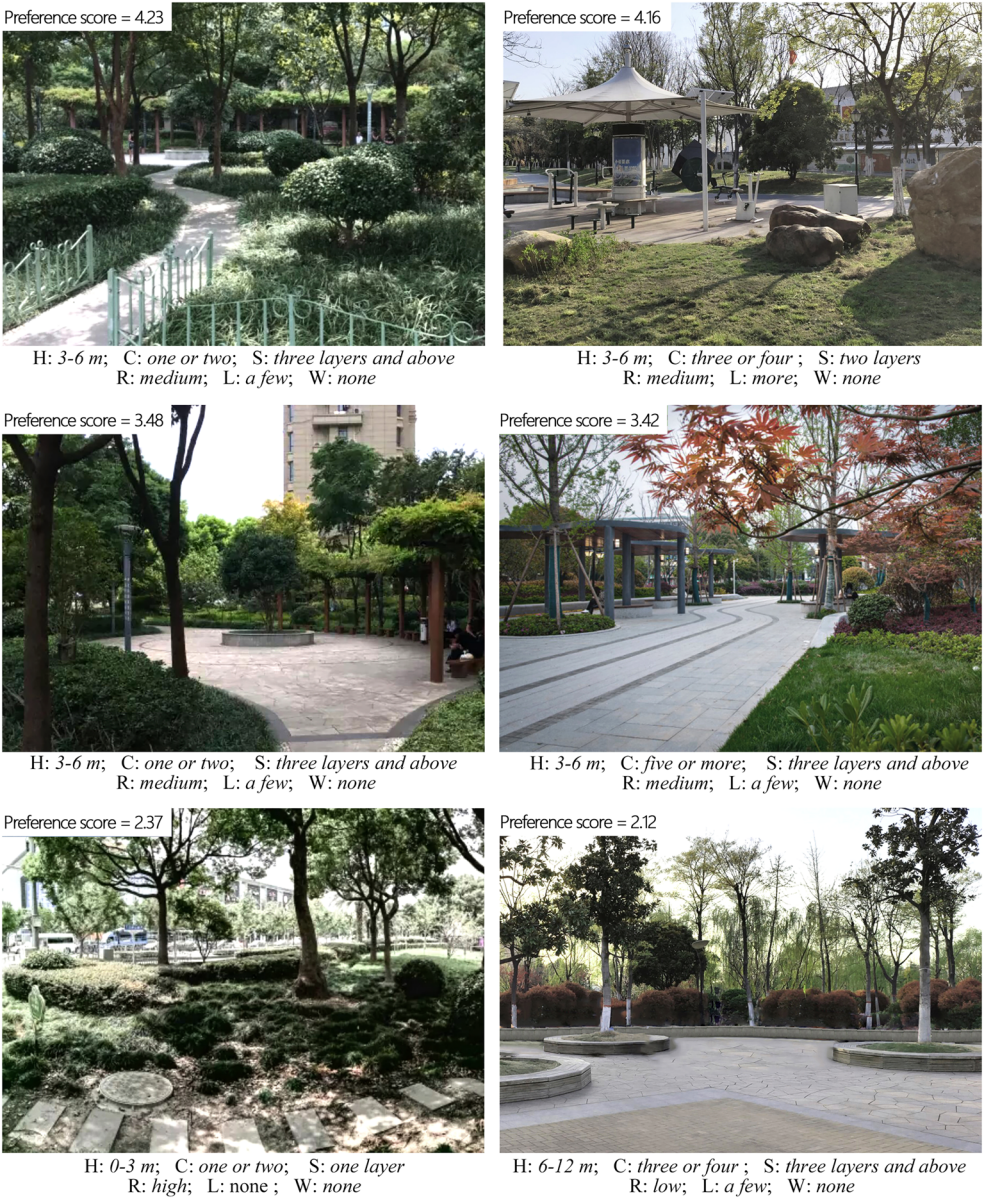
Photos with highest average score (top), photos with medium average score (middle), photos with lowest average score (bottom).

First, the intergroup reliability of the nine photos was tested. Using SPSS22.0, the reliability was calculated to be 0.825, displaying relatively high reliability. Accordingly, it could be concluded that the questionnaire survey was reliable and that the data obtained could be used for further detailed analysis. The mean score of each photo was denoted as S. For all the photos, the highest mean score was 4.23, and the lowest was 2.12 (overall scoring range: 1–5). The mean score of all the photos was 3.36. Figure 2 displays the two photos with the highest average score, the two photos with a medium average score, and the two photos with the lowest average score. In experiments where photos are used as a substitute for real landscapes, the average score of the photos can be considered effective to reflect the respondents’ visual impact assessment^56^.

### Demographic characteristics and visual impact assessment

To study the correlation between demographic characteristics and visual impact assessment, one-way ANOVA was first used to explore their relationship. As revealed by the calculation, a significant difference existed in the mean scores of respondents of different genders (F = 7.065, *p* = 0.001) and respondents of different residential types (F = 11.155, p < 0.001), but no significant difference was observed in the scores of respondents of different ages (F = 1.025, *p* = 0.521) and different education levels (F = 0.984, *p* = 0.662). Kendall correlation analysis was conducted to test the correlation between demographic characteristics and visual impact assessment. The calculation results indicated that the mean score of the photos (denoted as S) was correlated with gender (positively) and residential type (positively) but was not correlated statistically with age and education level (as shown in Table 5).

**Table 5:** The correlation between visual impact assessment and demographic variables.

		Score	Gender	Age	Education level
Gender	Coefficients	0.376**			
	Significance	0.002			
Age	Coefficients	0.076	0.048		
	Significance	0.753	0.835		
Education level	Coefficients	− 0.025	0.085	− 0.149	
	Significance	0.792	0.672	0.208	
Residential type	Coefficients	0.356**	0.172	0.105	0.205
	Significance	0.001	0.526	0.211	0.403

** Correlation is significant at the 0.01 level.

Multiple linear stepwise regression analysis was conducted to further study the data. In the multiple regression model, gender, age, education level, and residential type were taken as independent variables, and the mean score was set as the dependent variable. The analysis results indicated that gender and residential type exerted a significant influence on the respondents’ visual impact assessment, whereas the influence of age and education level was so weak that they could be excluded from the model (as shown in Table 6). The analysis also showed that the reciprocal effect among gender, age, education level, and residential type was relatively weak.

**Table 6:** Multiple linear stepwise regression analysis.

	Unstandardized coefficients		Standardized coefficients	t	Sig	Collinearity statistics	
	B	SE	Beta			Tolerance	VIF
(Constant)	4.356	0.376		13.458	0.000		
Gender	0.561	0.011	2.418	5.435	0.001	0.951	1.045
Residential type	0.245	0.051	0.451	3.57	0.016	0.945	1.748

Adjusted R^2^ = 0.321, n = 297.

To strengthen the reliability and objectivity of this model, it was necessary to test the normality of residual errors, variance analysis, and collinearity, which was completed by the Kolmogorov‒Smirnov test. As the results show, the residual errors were distributed normally (Kolmogorov‒Smirnov Z = 0.726, *p* = 0.835 > 0.05), and landscape features were linearly correlated with the standard deviation (F = 11.645, *p* = 0.000). According to Arriaza, et al.^12^, when the tolerance value is < 0.2 or the VIF is > 10, the model is problematic. Therefore, the model in this study was reliable, and no collinearity existed because the tolerance value was 0.865, which is larger than 0.2, and the VIF was 1.126, which is smaller than 10.

### The visual preference of different gender groups and landscape features

The mean preference scores for each photo by male elderly respondents and female elderly respondents were set as the dependent variables. The landscape features of the photos (height of trees, green colour richness, stratification of green landscapes, green space ratio, leisure facilities, and water landscape) were taken as the independent variables. As shown in the multiple linear stepwise regression model, the significant predictors for males and females were different (as shown in Table 7). For male respondents, the height of trees, green space ratio, and leisure facilities were reliable predictors. For female respondents, the height of trees, green colour richness, and stratification of green landscapes were reliable predictors.

**Table 7:** Linear regression analysis of the photos’ landscape features for different gender groups.

Dependent	Independent	Unstandardized coefficients	Standardized coefficients	t	Sig	Collinearity statistics	
		B	Beta			Tolerance	VIF
Scores for female respondents (R^2 = 0.628, N = 141)	Constant	1.855		2.418	0.015		
	Height of trees	0.527	0.598	2.657	0.011	0.898	1.243
	Green colour richness	0.278	0.425	1.563	0.021	0.963	1.981
	Stratification of green landscapes	0.872	0.525	2.235	0.018	0965	1.231
Scores for male respondents (R^2 = 0.681, N = 156)	Constant	1.537		3.698	0.035		
	Height of trees	0.962	0.963	2.121	0.001	0.898	1.128
	Green space ratio	0.165	0.515	1.654	0.011	0.813	1.064
	Leisure facilities	0.865	0.755	2.365	0.002	0.969	1.021

The K-S test was conducted to verify whether there was collinearity between the two models. As shown in Table 7, the residual errors were distributed normally (female: K-S Z = 0.963, *p* = 0.312; male: K-S Z = 0.815, *p* = 0.642). Therefore, it could be concluded that there was no collinearity between the two models.

### The visual preference of respondents with different residential types and landscape features

The mean preference scores for each photo by respondents who lived alone and those who lived with family were set as the dependent variables. The landscape features of the photos (height of trees, green colour richness, stratification of green landscapes, green space ratio, leisure facilities, and water landscape) were taken as the independent variables. As shown in the multiple linear stepwise regression model, the significant predictors for respondents who lived alone and those who lived with family were different (as shown in Table 8). For respondents who lived alone, the height of trees, green colour richness, and leisure facilities were reliable predictors. For those who lived with family, the green space ratio and leisure facilities were reliable predictors.

**Table 8:** The linear regression analysis of the photos’ landscape features for respondents with different residential types.

Dependent		Unstandardized coefficients	Standardized coefficients	t	Sig	Collinearity statistics	
		B	Beta			Tolerance	VIF
Scores for respondents who live alone (R^2 = 0.465, N = 125)	Constant	1.797		3.345	0.002		
	Height of trees	0.434	0.417	2.452	0.012	0.825	1.141
	Green colour richness	0.535	0.542	3.345	0.025	0.854	1.222
	Leisure facilities	0.556	0.341	2.845	0.005	0.953	1.054
Scores for respondents who live with family (R^2 = 0.538, N = 172)	Constant	1.357		3.451	0.005		
		0.545	0.452	2.143	0.001	0.985	1.221
	Leisure facilities	0.442	0.322	2.245	0.012	0.925	1.126

The K-S test was conducted to verify whether there was collinearity between the two models. As shown in Table 7, the residual errors were distributed normally (respondents who lived alone: K-S Z = 0.955, *p* = 0.158; respondents who lived with family: K-S Z = 0.856, *p* = 0.216). Therefore, it could be concluded that there was no collinearity between the two models.

## Discussion

### The influence of demographic characteristics on visual impact assessment

The research results indicate that different demographic characteristics can lead to different visual preference evaluations of the same set of pictures among elderly individuals, including gender and living arrangement type.

As revealed in this study, compared with elderly men, elderly women provided lower visual assessments. Specifically, the mean score of female respondents was 3.5, while the mean score of male respondents was 3.28. Although Yao, et al.^55^ maintained that gender did not influence the visual impact assessment of landscapes, the results obtained in this study are contrary to those findings. In this study, gender differences led to different visual impact assessments of landscapes, which is basically consistent with the conclusion of Richardson and Mitchell^57^. This result can be explained by the theory of evolutionary stasis^58^. In the long history of evolution, women have shown a strong interest in plants with rich colours. This may be related to the fact that women often play the role of food pickers, and colourful plants are usually rich in vitamins, minerals and other nutrients, which is important for women to produce healthy offspring^16^ However, colourful plant landscapes are not common in urban green spaces such as pocket parks, which partly explains why older women have lower scores for pocket park landscapes than older men.

Previous studies have suggested that age is an important factor that affects elderly respondents’ visual impact assessments. For example, Berg and Koole^59^ found that age was negatively correlated with visual impact assessment in their study of the visual impact assessment of water landscapes. Howley, et al.^60^ observed that age was positively correlated with visual impact assessments in their study of traditional farm landscapes. However, the results of this study indicated that age was not a significant factor that influenced elderly people’s visual impact assessment of pocket park landscapes. In previous studies, the physical characteristics of each age group differed greatly, whereas the research object of this study was elderly individuals who were divided into three age groups, namely, 60–69 years old, 70–79 years old, and 80 years old and above. Their physical characteristics were similar or not drastically different, and they had similar needs for pocket park landscapes and shared common standards for the visual impact assessment of landscapes.

Svobodova, et al.^61^ and Wang, et al.^62^ maintained that education level significantly influences people’s visual impact assessment. For example, highly educated respondents prefer natural vegetation that is closely associated with ecological interests. However, the results of this study are contrary to their findings. This study found that the influence of education level on elderly respondents’ visual impact assessment of pocket park landscapes was not significant. No remarkable difference was observed between groups with different education levels, which is in line with the conclusions of Molnarova, et al.^63^ and Zhen, et al.^64^.

This study found that residential type exerted an impact on elderly people’s visual impact assessment of pocket park landscapes. Overall, elderly people who lived alone provided lower scores than those living with family. Specifically, the average score of elderly people who lived alone was 3.08, whereas the average score of those who lived with family was 3.17. The difference may be justified by the fact that elderly individuals who live alone have a greater ability to take care of themselves. Compared with those who live with families, they are more sensitive and observant to their surroundings^65^. Accordingly, they may have higher expectations for pocket park landscapes. If the actual landscapes cannot meet their expectations, they may be likely to provide lower scores.

Overall, no consensus has been reached regarding the influence of elderly individuals’ gender, age, educational level, and residential type on their visual impact assessment. Therefore, more efforts must be made to explore the influence of elderly people’s demographic characteristics on their visual impact assessment of pocket park landscapes.

### The interaction between different demographic characteristics

The main reason why there is still no definite conclusion about the influence of demographic characteristics on the visual impact assessment of the landscape environment is the interaction between the demographic characteristic variables. To date, there has been no concrete conclusion on the working mechanism or range of this interaction. This is mainly because the respondents are from different countries and regions and therefore differ in their cultural background and personal experiences. Accordingly, to judge whether there is interaction between the variables, a regression model must be used. In this experiment, a multiple linear regression model showed that the interaction between gender, age, education level and residential type was relatively weak (as shown in Table 5). This may be because the location of this experiment was Nanjing, Jiangsu Province, China, and the respondents were all Chinese nationals. In an area with large population mobility such as Nanjing, the interaction between gender, age, education level, and residence type is unstable. In experimental research, conclusions from other articles should not be simply quoted but should be recalculated in the specific regional and cultural context to ensure the objectivity of the experiment^66,67^.

### Demographic characteristics and landscape features

#### The visual preference of different gender groups and landscape features.

Gender differences influence elderly people’s visual impact assessments of pocket park landscapes. As revealed in this study, trees with a height of 3 to 6 m, medium green space ratio, and more leisure facilities have a significant effect on improving elderly men’s visual impact assessment, while elderly women’s visual demand can be satisfied by the following landscape specifications: the height of trees should be 0–3 m, the number of colours should be more than five, and the landscape should be three-layered or more.

Elderly men enjoy engaging in leisure activities such as sunbathing, chatting, playing chess, jogging, and practicing Tai Chi in the pocket park^68^. These activities have a demand for hard paving, which can more easily meet the needs of elderly men to use the park than green ground coverings. Trees with a height of 3–6 m can provide a good shading effect while facilitating the activities of elderly men, similar to the roof of a pocket park, providing them with shade in summer and shelter in winter. In addition, elderly men are more likely to participate in sports activities because of social factors, and more leisure facilities provide a social gathering place for the elderly men^33^. To some extent, this explains why elderly men prefer 3- to 6-m-high greening, a medium proportion of green space, and pocket park landscapes with more leisure facilities. This finding is consistent with the studies of Yücel^69^ and Robinson^70^.

Compared with elderly men, elderly women pay more attention to the need for safety^71^. Trees with a height of 0–3 m and three or more layers of landscape can form a single-sided and double-sided shelter, creating a good sense of spatial boundary. This can isolate noise, form a semiprivate space, and enhance the sense of security of elderly women. In addition, trees with a height of 0–3 m are in line with the scale of Chinese gardens, making people feel more intimate. Compared with the single-layer landscape of pure forest or pure grass, a greening landscape structure of three layers or above can maintain and improve the vigorous vitality of pocket park greening. Women's desire for beauty leads them to prefer pocket park landscapes with five or more colours, possibly because the richer the colour of plants is, the more they can satisfy people’s pursuit of beauty^15^.

#### The visual preference of elderly groups with different residential conditions and landscape features

The residential type of the elderly respondents also affected their visual impact assessment of pocket park landscapes. As can be concluded from this study, elderly people who lived with their family preferred pocket park landscapes with a medium green space ratio and more leisure facilities. In contrast, people who lived alone preferred landscapes with trees that are 0–3 m high, five or more colours, and medium leisure facilities.

In China, the task of taking care of children usually rests with elderly individuals, especially those who do not live alone^72^. They also need to take care of young children when they exercise or socialize in pocket parks. A higher number of leisure facilities can not only provide them with places for exercise and communication but can also meet their need to care for young children and have fun with people of different ages. Pocket parks with a medium proportion of green space can provide soft grass that can ensure the safety of children's play, while hard pavement can provide a playground for elderly individuals. In addition, elderly individuals who do not live alone usually assume the responsibility of taking care of their spouses^73^. If they are unable to move, flat rigid squares can weaken the resistance of elderly couples to use pocket parks together. To some extent, this explains why elderly people who do not live alone prefer pocket park landscapes with a medium proportion of green space and more leisure facilities.

Due to the decline of elderly people’s visual function and the tedium and loneliness of retirement life, elderly individuals, especially those who live alone and still have certain viability, hope to be able to integrate into the community and to be recognized and accepted. Due to fear of ageing or loss of ADL function^74^, elderly individuals who live alone are likely to engage in antiaging activities and to prefer diverse and bright colours. Greening with five or more colours can not only stimulate their reduced senses but can also contribute to a vigorous and unrelenting mentality^75^. With regard to the selection of activity places, elderly people prefer secluded places with lush trees that are not easily disturbed by sight. The activity scale is relatively small and is usually limited to 2–4 people sitting idly to chat. Through communication and interaction with others, they can release their feelings and emotions. Therefore, pocket park landscapes with trees that are 0–3 m high, five or more colours, and medium leisure facilities are more attractive to elderly people who live alone.

### Limitations and future research directions

1. The selection of plant species is one limitation. In this study, only common shrubs and trees in field photos were used as landscape parameters for factor intervention. Although this method has been widely used by previous researchers^47,76^, it fails to fully reflect the diversity of plant species in parks in subtropical monsoon regions^77^.
2. The photos in this paper were all taken in spring. Although these photos represent the landscape characteristics of most seasons of the year under the climatic conditions in Nanjing, they fail to fully reflect the landscape characteristics throughout the year, especially in winter.
3. This study did not take into account that respondents may have different landscape preferences in different seasons. Future studies can further explore the impact of seasonal factors on preferences to more comprehensively assess respondents' preferences for pocket park landscapes.
4. In the questionnaire, the frequency of respondents' use of pocket parks was not assessed, which may have a potential impact on the results. For example, respondents who use parks more frequently may evaluate the landscape characteristics of parks more positively^71^. Therefore, in a follow-up study, it is suggested to further explore the relationship between the frequency of pocket park use and landscape preference.

## Conclusion

As elderly people’s physical function declines, their retirement life is rather limited and their activity scope is small. In this sense, pocket parks have become main sites of leisure activities for elderly people in cities. Improving the quality of pocket park landscapes can contribute to the psychological recovery of elderly people and delay body function decline, which in turn improves the quality of their later life. When designing a pocket park landscape suitable for elderly people, designers need to understand the demands of elderly people with different demographic characteristics and design more desirable pocket park landscapes accordingly.

Starting from the relationship between the landscape features of pocket parks and elderly people’s visual impact assessment, this study found that elderly people of different genders and different residential types tend to choose different pocket park landscapes. Elderly men prefer pocket park landscapes with 3- to 6-m-high trees, medium green space ratio, and more leisure facilities, while elderly women are in favour of pocket park landscapes with 0- to 3-m-high trees, five or more colours, and three or more layers. Elderly people who live with their family prefer pocket park landscapes with a medium green space ratio and more leisure facilities, while elderly people who live alone prefer pocket park landscapes with trees that are 0–3 m high, five or more colours, and medium leisure facilities. This study has reference significance and value for the future construction of urban pocket parks and can provide insight for future researchers.

## Institutional review board statement

The work was approved by the Committee of China University of Mining and Technology.

## Informed consent statement

Informed consent was obtained from all subjects involved in the study.

## Data Availability

The datasets used and/or analysed during the current study are available from the corresponding author on reasonable request.
